# SNP-based analysis reveals unexpected features of genetic diversity, parental contributions and pollen contamination in a white spruce breeding program

**DOI:** 10.1038/s41598-021-84566-2

**Published:** 2021-03-02

**Authors:** Esteban Galeano, Jean Bousquet, Barb R. Thomas

**Affiliations:** 1 Department of Renewable Resources, 442 Earth Sciences Building, University of Alberta, Edmonton, AB T6G 2E3 Canada; 2 Department of Wood and Forest Sciences, Pavillon Charles-Eugène Marchand, Université Laval, Quebec City, QC G1V 0A6 Canada

**Keywords:** Plant breeding, Genetic markers, Plant sciences

## Abstract

Accurate monitoring of genetic diversity levels of seedlots and mating patterns of parents from seed orchards are crucial to ensure that tree breeding programs are long-lasting and will deliver anticipated genetic gains. We used SNP genotyping to characterize founder trees, five bulk seed orchard seedlots, and trees from progeny trials to assess pollen contamination and the impact of severe roguing on genetic diversity and parental contributions in a first-generation open-pollinated white spruce clonal seed orchard. After severe roguing (eliminating 65% of the seed orchard trees), we found a slight reduction in the Shannon Index and a slightly negative inbreeding coefficient, but a sharp decrease in effective population size (eightfold) concomitant with sharp increase in coancestry (eightfold). Pedigree reconstruction showed unequal parental contributions across years with pollen contamination levels between 12 and 51% (average 27%) among seedlots, and 7–68% (average 30%) among individual genotypes within a seedlot. These contamination levels were not correlated with estimates obtained using pollen flight traps. Levels of pollen contamination also showed a Pearson’s correlation of 0.92 with wind direction, likely from a pollen source 1 km away from the orchard under study. The achievement of 5% genetic gain in height at rotation through eliminating two-thirds of the orchard thus generated a loss in genetic diversity as determined by the reduction in effective population size. The use of genomic profiles revealed the considerable impact of roguing on genetic diversity, and pedigree reconstruction of full-sib families showed the unanticipated impact of pollen contamination from a previously unconsidered source.

## Introduction

Forest companies implement tree breeding programs to increase volume in less time while ensuring that enough genetic diversity is maintained through successive breeding cycles to limit inbreeding when selecting superior material for deployment^1^. Genetic diversity must also be maintained in the breeding population, often at higher levels than in the production (seed) orchards, to allow for several generations of breeding to occur without impacting inbreeding levels^2^. The breeding population is the base for long term breeding, usually separated physically from the seed orchards, and maintained in a breeding arboretum (aka a clone bank). On the other hand, seed orchards are considered the “output delivery system” of the breeding population, with the very best individuals (best breeding values) present in the seed orchard^2^. The seed orchard needs to have a sufficient number of trees in the initial establishment so it can withstand roguing and maintain diversity during the lifetime of the orchard. White spruce (*Picea glauca* (Moench) Voss) is a transcontinental boreal species, with a typical optimal rotation age between 90 and 110 years^3,4^. These long rotations have led some companies to favour backward over forward selection strategies to realize faster gains in their white spruce programs by roguing rather than developing a new second-generation seed orchard which can take years to produce its first seed crop^5^. Progeny trial data is used to guide roguing of the seed orchard to obtain seed with higher gain for deployment sooner^5^. Meanwhile, building the next-generation orchard can be produced by crossing the best genotypes (based on progeny trial data), and grafting them into a higher gain second generation orchard, while waiting several years to obtain seed^5^. Ultimately, designing and establishing a superior elite breeding strategy for white spruce through controlled crosses has not been widely adopted^6^, resulting in seed orchards that are nearly identical to the breeding population.

As typically observed in many tree improvement programs, orchard managers use the number of cones, seeds and pollen produced in orchards to estimate diversity parameters, parental contributions and pollen contamination to obtain government approvals for the genetic gain and genetic diversity of reforestation stock^7,8^. However, the range in those parameters can vary considerably between seedlots, genotypes and taxa. In conifers, studies have shown that between 34 and 52% of parents usually contribute 80% of the progeny produced from a seed orchard seedlot^9–14^. Calculations, based on pollen flight monitoring in Alberta, have shown pollen contamination levels range between 9 and 100% across several first-generation white spruce seed orchards^15^. Furthermore, pollen production, conelet receptivity and pollination typically occurs over approximately a two-week period, when the prevailing wind direction can cause pollen contamination levels to increase inadvertently^8,16^. Tree improvement programs often have little to no strategy for pollen management using silvicultural practices, since local orchard pollen production is believed to be sufficient to both swamp pollen from external sources and account for the high seed yields found during mast years in white spruce^15^. However, these assumptions appear to be underestimating the impact of contamination on the genetic worth of the production seedlots we studied.

In recent years, high-throughput genotyping technology, relying on single nucleotide polymorphisms (SNPs), has proven to be a reliable methodology to estimate genetic diversity parameters of seedlots in tree breeding programs, including the Shannon Index, expected and observed heterozygosity, inbreeding coefficients, allele frequencies, coancestry (complete seedlot and tree pairs), and genotype-based pedigree reconstruction^17–20^. In addition, genotyping allows for more precise estimations of heritability and genetic gain compared with using pedigree information alone^13,17,21–23^. In white spruce, such genotyping approaches have enhanced the evaluation of allelic diversity in selected seedlots from seed orchards compared to natural populations^24^ and allowed for pedigree reconstruction^25^, genomic selection^17,26^, and traceability in breeding and propagation operations^27^.

To date, only a limited number of studies have individually assessed the impact of severe seed orchard roguing on diversity, parental contributions, pollen contamination and trade-offs with genetic gain in a conifer breeding program using a broad genetic marker approach^28,29^, while none have collectively addressed all of these issues. The present work was undertaken to consider the additional benefits of incorporating genomic tools into the evaluation of severe roguing on a conventionally managed first generation clonal white spruce orchard in central Alberta. The specific goals of the project were to: (1) evaluate the impact of a severe roguing on the effective population size and other diversity parameters of orchard seedlots produced in different years; (2) assess the imbalance of contributions among parents before and after severe roguing; (3) through pedigree reconstruction, obtain precise estimates of pollen contamination levels of seed orchard seedlots and families from a progeny trial associated with the breeding program; and (4) compare the methods used to estimate the effective population size and levels of pollen contamination.

## Results

### Genetic diversity and effective size of founders, seed orchard seedlots and progeny trials before and after roguing

**Figure 1:**
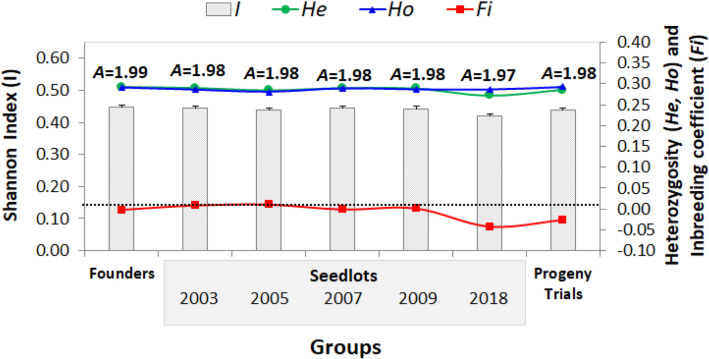
Genetic diversity parameters across groups (founders, seed orchard seedlots and progeny trials) and years in the G1 white spruce orchard, using tree genomic profiles with a set of 2000 SNPs. Bars indicate Shannon Index (*I*) + 1 standard error, and the green (dots), blue (triangles) and red (squares) lines indicate expected heterozygosity (*H*_*e*_), observed heterozygosity (*H*_*o*_) and the inbreeding coefficient (*F*_*i*_), respectively. *A* is the average number of alleles per SNP. The black dotted line denotes a zero inbreeding coefficient.

Overall genetic diversity was found to reside within (98%) and not between (3%) the different groups under study (founders, seedlots, progeny trials) (*Φ*_*PT*_ = 0.028, *P* < 0.001) (Supplementary Table S2). Observed heterozygosity (*H*_*o*_) ranged between 0.286 (seedlot 2003) and 0.292 (founders) with a mean of 0.288 (Fig. 1), and expected heterozygosity (*H*_*e*_) ranged between 0.273 (seedlot 2018, after final roguing) and 0.291 (founders) with a mean of 0.286 (Fig. 1). The Shannon Index (*I*) ranged between 0.42 (seedlot 2018) and 0.45 (founders) (mean 0.44), the average number of alleles per SNP ranged between 1.97 for seedlot 2018 (after roguing), to 1.98 for other seedlots and 1.99 for founders (Fig. 1). The inbreeding coefficient (*F*_*i*_) ranged between − 0.042 (seedlot 2018) and 0.01 (seedlot 2005) (mean − 0.008) (Fig. 1). Observed heterozygosity estimates from seedlots 2003–2009 were close to the Hardy–Weinberg expectation (*H*_*o*_ = *H*_*e*_*, F*_*i*_ = 0), but after roguing, the 2018 clonal seed orchard lot exhibited an excess of observed heterozygotes leading to a slightly negative inbreeding coefficient (*F*_*i*_ =  − 0.042). The orchard seedlots, as a whole, showed no sign of inbreeding given that inbreeding coefficient values were near or below zero.

Effective population size (*N*_*e*_), calculated based on the number of cones versus estimates based on SNPs, presented the lowest values of 18 and 12, respectively, after roguing (seedlot 2018). Seedlots from 2007 and 2003 showed the highest *N*_*e*_ values with 51 and 166, based on cones versus SNPs, respectively (Table 1).

**Table 1:** Summary table with population size and coancestry parameters across groups (founders, seed orchard seedlots by year of collection and progeny trial trees) in the G1 white spruce orchard, using tree genomic profiles with a set of 2000 SNPs.

	Groups						
	Founders	2003 seedlot	2005 seedlot	2007 seedlot	2009 seedlot	2018 seedlot	Progeny trials**
**Parameter**							
*N* (number of genotypes)	151	151	151	151	151	53	151
*N*_*e*_ (using number of cones*)	n/a	48.6	35.3	51.4	46	18	n/a
*N*_*e*_ (using genomic profiles)	500	166	59	158	96	12	31
*N*_*ratio*_	3.3	1.1	0.4	1.0	0.6	0.2	0.2
*Ɵ*	0.001	0.003	0.008	0.003	0.005	0.040	0.016
**Relatedness level**							
Unrelated (%)	67.2	74.4	73.8	74.8	74.2	52.4	76.5
2nd cousins (%)	31.5	18.8	13.3	17.0	16.4	8.7	11.7
1st cousins (%)	1.3	0.6	1.1	0.8	0.8	4.0	1.0
Half-sibs (%)	0.0	5.9	10.9	7.2	8.0	32.4	10.2
Full-sibs (%)	0.0	0.3	1.0	0.3	0.5	2.5	0.6

*N* is the number of genotypes present in the clonal seed orchard (actual population size). Effective population size (*N*_*e*_) was calculated by two methods: using numbers of cones per genotype and SNP profiling (see Methods). *N*_*ratio*_ is *N*_*e*_*(genomic profiles)/N*. Coancestry coefficient (*Ɵ*) was disaggregated to calculate relatedness levels (%) for each group.

*These values were calculated following FGRMS (2016).

**Relatedness levels for progeny trials were calculated among families.

In contrast, the coancestry coefficient showed its highest value of 0.040 in the 2018 seedlot after roguing, representing an 8- and 40-fold increase when compared to the value of the 2009 seedlot prior to roguing and that of founders, respectively (Table 1). In addition, the highest levels of relatedness were found after roguing, with 2.5% full-sibs, 32% half-sibs and 52.4% unrelated, when compared with seedlots before roguing, with 0.3–1.0% full-sibs, 6–11% half-sibs and 74% unrelated (Table 1). Although the founders showed no presence of half- or full-sibs, 31.5% were second cousins while most of the remaining trees were unrelated (67%) (Table 1). Progeny trial trees showed similar levels of relatedness among families when compared to orchard seedlots (Table 1). However, levels of relatedness of individuals within putative half-sib families showed varying levels among families, ranging between 1 and 12% for full-sib individuals, 73–89% for half-sib individuals (compared to 100% expectation), and 0–11% for unrelated individuals (Supplementary Table S3).

### Parental contributions in the seed orchard before and after roguing

**Figure 2:**
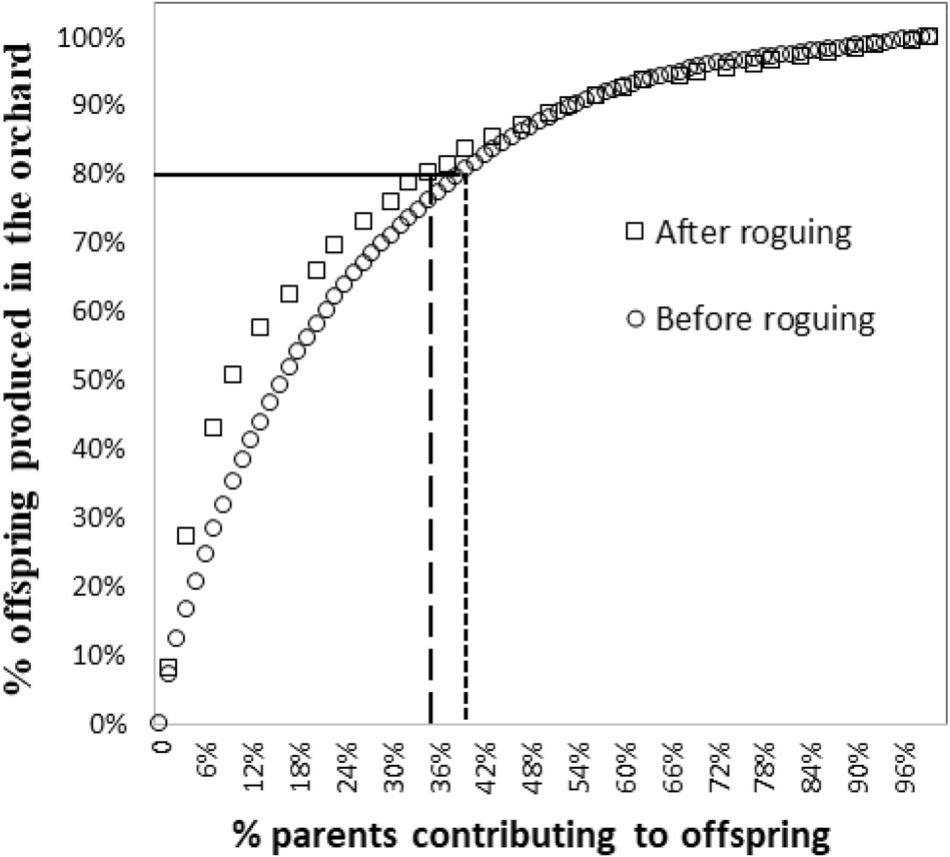
Cumulative reproductive success in the G1 white spruce clonal seed orchard obtained using pedigree reconstruction with genomic profiles with a set of 2000 SNPs. Circles represent the reproductive success rate of 151 parents over 420 offspring from five seedlots obtained before roguing the orchard. Squares represent the reproductive success rate of 53 parents over 105 offspring from 1 seedlot obtained after roguing the orchard. The line represents 80% of the offspring produced in the orchard, corresponding to a % parents before (short dashed lines) and after (long dashed lines) roguing.

**Figure 3:**
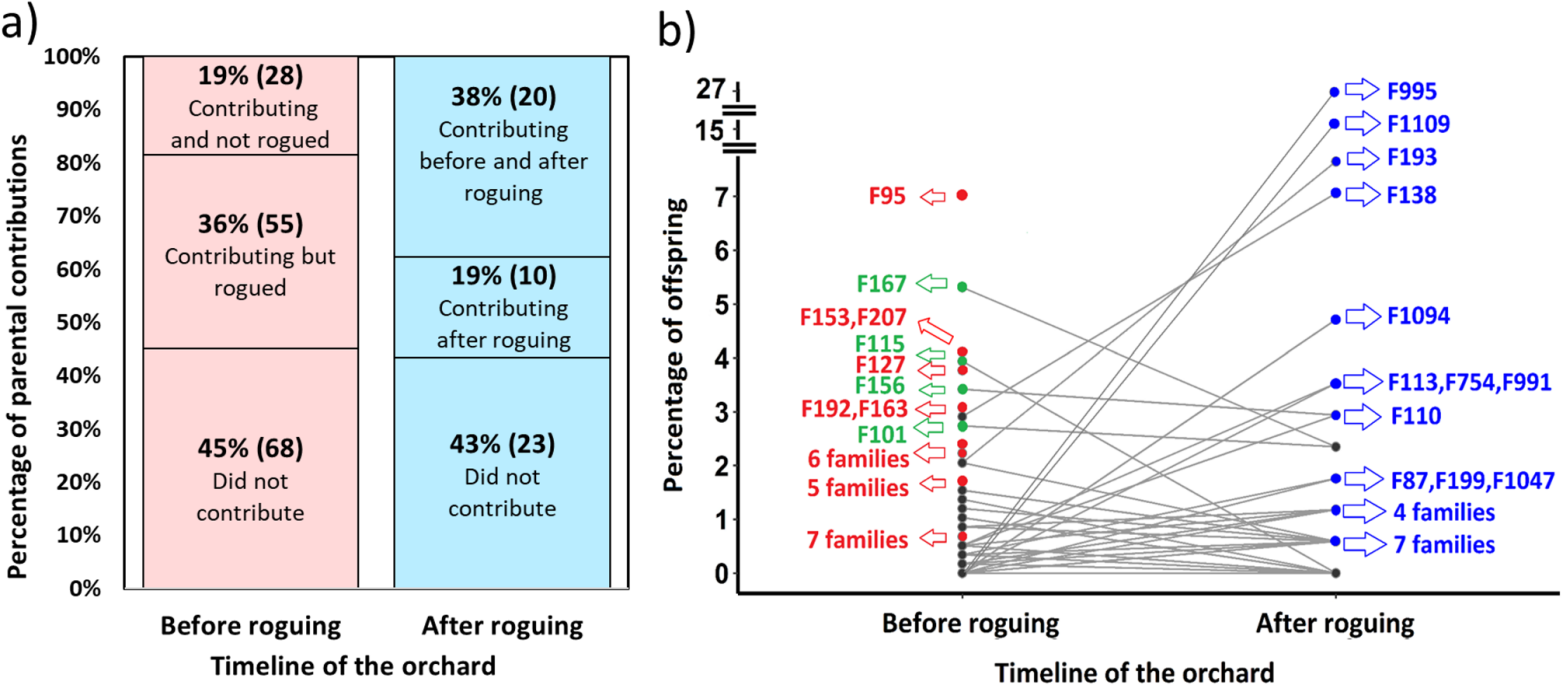
Changes in parental contributions before and after roguing the G1 white spruce clonal seed orchard estimated with pedigree reconstruction using genomic profiles with a set of 2000 SNPs. (**a**) Stacked bar chart (100% baseline) showing changes in parental contributions influenced by genotype roguing; (**b**) Rank-order line chart showing changes in specific genotype contributions (100% baseline), with some genotypes contributing and others eliminated from the G1 clonal seed orchard (red dots), some genotypes decreasing their contribution after roguing the clonal seed orchard (green dots) while other genotypes increasing their contribution after roguing (blue dots). Calculations before roguing were based on 420 seedlings and 151 parents, and calculations after roguing were based on 105 seedlings and 53 parents.

By performing the parental assignment using genomic profiles, we confirmed that all offspring from the seedlots and in the progeny trials originated from the orchard, with significant differences in parental contributions including external pollen levels (Table 2). For 80% of the offspring produced in the orchard, we observed a decrease from 39 to 35% of the orchard parents’ contributions before and after roguing, respectively (Fig. 2). In contrast, 45% and 43% of the total number of parents from the orchard did not contribute to the seedlot offspring, either before or after roguing, respectively (Fig. 3a). From the total number of parents siring offspring before roguing, 19% were retained in the orchard, while 36% were removed (Fig. 3a). For the seedlot produced after roguing (2018), 38% of parents continued to contribute and 19% of the parents (10 genotypes) began contributing for the first time (Fig. 3a).

**Table 2:** Summary table for parental contributions (sex1, sex2, both sexes) for five seedlots collected at different years from the G1 white spruce orchard.

Parameter	Contribution per year of crop				
	2003	2005	2007	2009	2018
**Parents (sex1)**					
No	28	23	35	38	28
%	18	15	23	25	53
**Parents (sex2)**					
No	27	32	36	40	8
%	18	21	24	26	15
**Parents (both sexes)**					
No	43	38	53	61	30
%	28	25	35	40	57
**Pollen contamination (%)**					
Using traps* (%)	100	23	11	10	n/a
Using SNPs (%)	51	26	28	12	18
Wind direction (average)**					
Degrees (°)	236	201	223	191	201
**Days with west wind direction**					
No	11	6	7	8	6

Calculations were done using pedigree reconstruction using genomic profiles with a set of 2000 SNPs. Calculations were based on two different pedigree reconstructions: 420 seedlings and 151 parents for 2003, 2005, 2007 and 2009 (years before roguing), and 105 seedlings and 53 parents for 2018 (year after roguing). Pollen contamination (%) was calculated using two methods (traps and SNP profiling), wind direction in degrees and number of days with a west wind direction out of 15 days of assessment for each year.

*Calculated following FGRMS (2016).

**Calculated using the weather database from ‘Grande Prairie A’ station (https://climate.weather.gc.ca/).

Surprisingly, of the 55 genotypes contributing to each seedlot assessed before roguing (2003 to 2009 seedlots), six genotypes were rogued (F95, F153, F207, F127, F192, F163 (Fig. 3b, red circles). Furthermore, 23 genotypes increased their parental contribution after roguing (Fig. 3b, blue circles), with some notable parental contributions, F995 (increasing from 0 to 27%) and F1109 (increasing from 0 to 15%). Before roguing (2003–2009 seedlots), parents from both sexes (sex1 and sex2) were contributing equally to each seedlot. However, after roguing (2018 seedlot), sex1 parents increased their contribution to 53% while sex2 parents decreased to only 15% (eight parents) (Table 2). Using pedigree reconstruction, seven full-sib families were shown to have the highest contributions in seedlots for all five years of sampling (Supplementary Fig. S1).

### Pollen contamination in the seed orchard seedlots and progeny trials

Levels of pollen contamination in the clonal seed orchard, defined as the proportion of trees outside the orchard siring the progeny, were reported to be between 10 and 100% using pollen traps^15^, and between 12 and 51% using genomic profiles (SNPs) (Table 2), without a statistically significant correlation in response across years between the two methods (*r* = 0.80, *P* > 0.01) (Supplementary Fig. S2). For example, for the 2003 seedlot, the levels of pollen contamination were estimated to be 100% and 51% based on pollen traps versus SNPs, respectively, and for the 2007 seedlot, contamination levels were estimated at 11% and 28% based on pollen traps versus SNPs, respectively (Table 2).

### Correlations for ***N***_***e***_ and levels of pollen contamination between number of cones and SNPs

**Figure 4:**
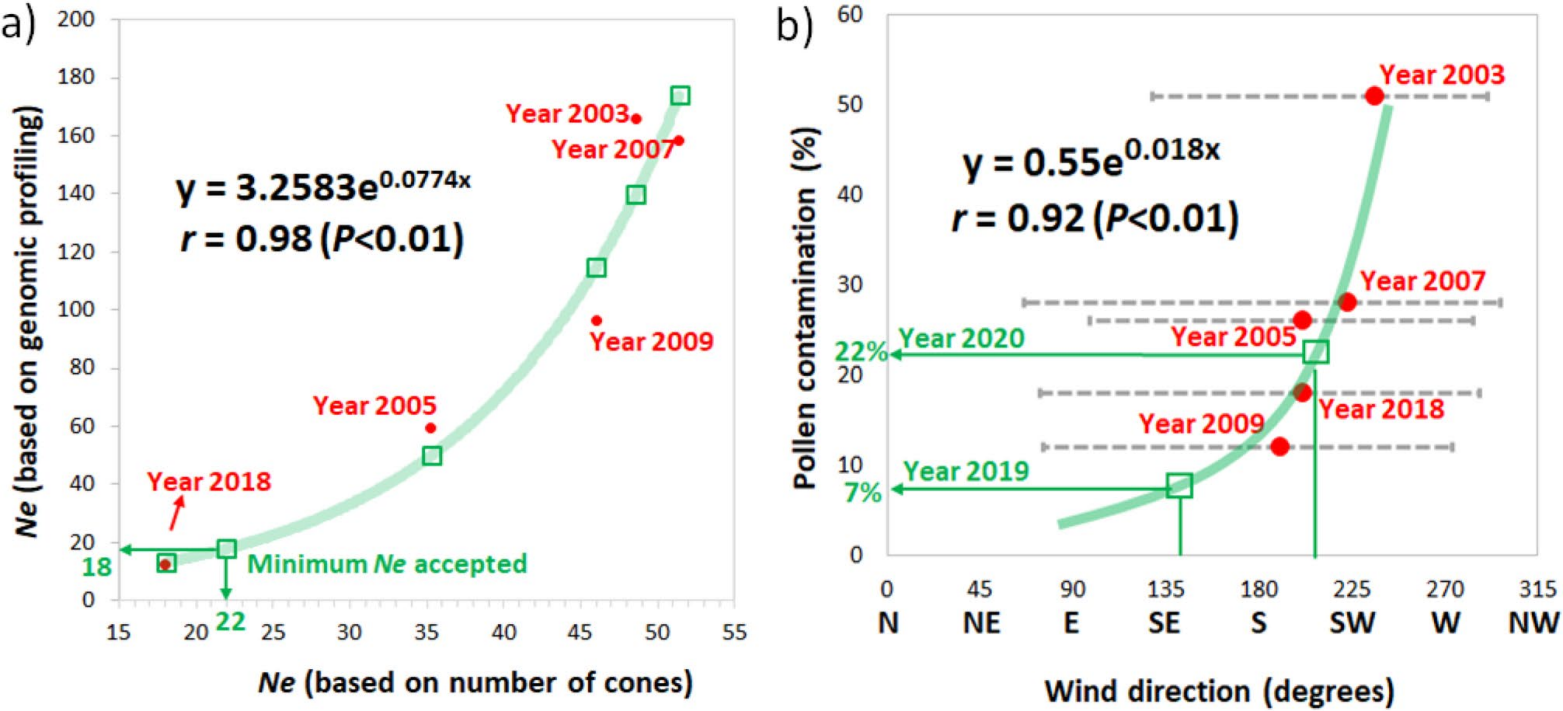
Correlation plots (Pearson’s) showing exponential trendlines (green lines), equations, R-squared values and *P* values for different traits. (**a**) Effective population size (*N*_*e*_) calculated using the number of cones per genotype versus using genomic profiles; (**b**) Pollen contamination levels (%) estimated using SNP profiling versus wind direction average between 15 and 31 May of each year. Red dots show data used for fitting the equations. Green squares show predicted values using the equations. Grey dotted lines indicate all values, including maximum and minimum wind speed values. Weather data for 2003–2018 was obtained from www.climate.weather.gc.ca, and for 2019–2020 was obtained from www.acis.alberta.ca. The “Minimum *N*_*e*_ accepted” corresponds to the value suggested in the Alberta forestry policies, which is *N*_*e*_ = 18^8^.

Effective population size calculations using the number of cones per genotype showed a high correlation with values calculated using genomic profiles (SNPs) (*r* = 0.98, *P* < 0.01) (Fig. 4a). However, the fitted exponential relationship (Fig. 4a) also showed that an *N*_*e*_ = 22 calculated using cones per genotype corresponded to an *N*_*e*_ = 18 using SNPs. Levels of pollen contamination estimated with genomic profiles showed no correlation with temperature, precipitation, relative humidity, or wind speed (Supplementary Fig. S3); however, a strong correlation was found with wind direction during the pollen flight/receptivity time period (*r* = 0.92, *P* < 0.01) (Fig. 4b). The fitted exponential equation (Fig. 4b) predicted levels of pollen contamination of approximately 7% and 22% for years 2019 and 2020, respectively, for the G1 clonal seed orchard (Fig. 4b). However, further SNP profiling needs to be performed for those years to confirm these predictions.

### Pollen contamination in progeny trials

**Figure 5:**
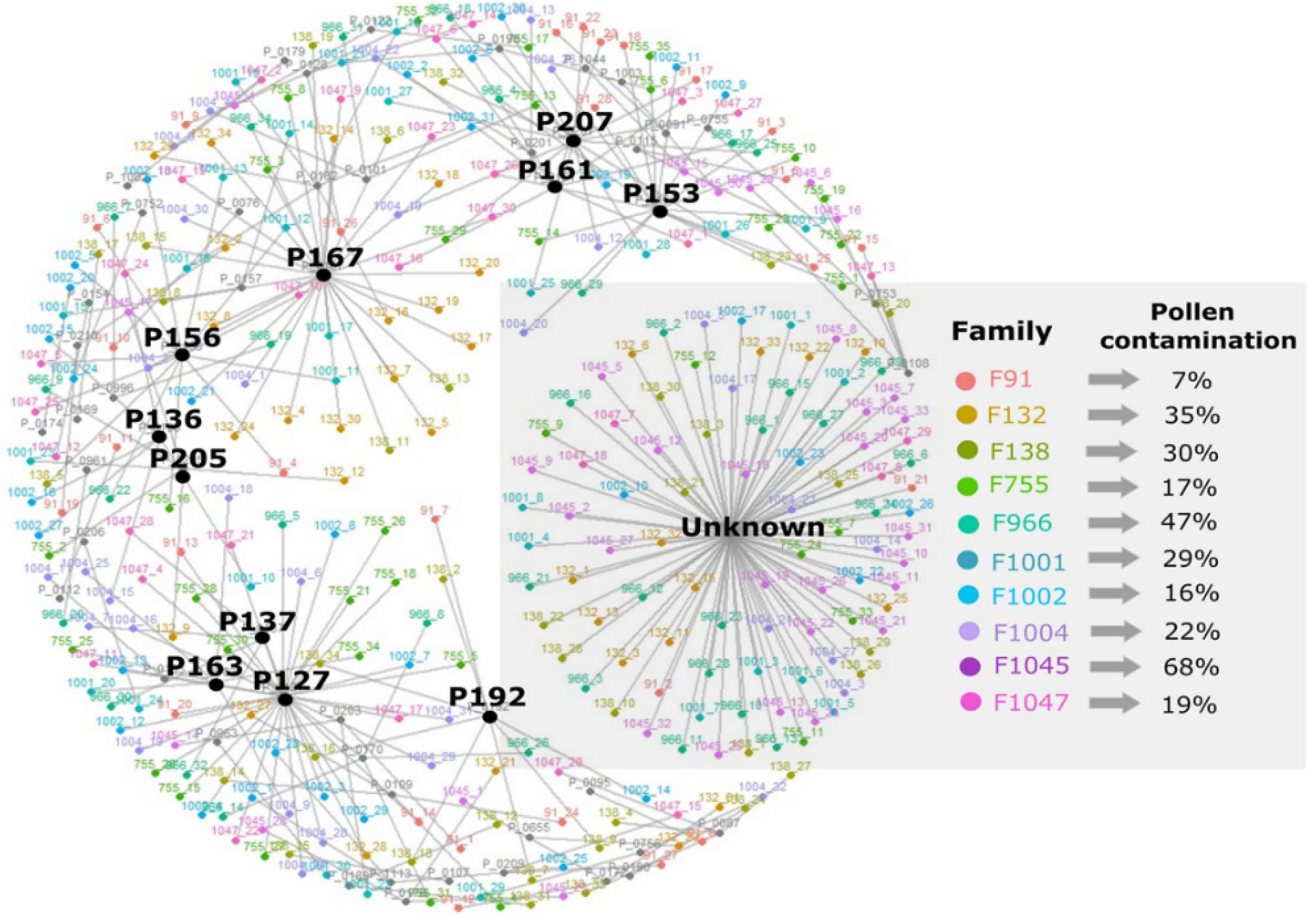
Network diagram representing the pedigree reconstruction of 10 families (328 trees) from a 15-year-old progeny trial (two sites) associated with the Region G1 white spruce program. Using 2000 SNPs, paternal (P, large black dots) assignment was achieved using CERVUS and COLONY software with the 10 known mothers (small coloured dots). For each of the 10 families (F), the per cent (%) pollen contamination from all unknown male contributions is highlighted in the grey square.

We reconstructed the pedigrees of 10 open-pollinated families, originating from parent trees in the clonal seed orchard, from two progeny trials associated with the G1 white spruce program, from approximately 33 trees per family, to assess the level of pollen contamination within each family which ranged from 7% (F91) to 68% (F1045) (Fig. 5). Overall, 11 fathers (Fig. 5) and only seven crosses (Supplementary Fig. S4) were found to be contributing to more than five offspring in each family, among all 328 progeny trees genotyped. Thirty percent (90 trees) of the progeny were from unknown fathers (Fig. 5).

## Discussion

### Impact of severe roguing on genetic diversity and coancestry

As expected, most alleles at biallelic SNP loci were detected across all seedlots generated from the G1 white spruce breeding program, with a decrease of only 1% in the seedlot after the sever roguing compared to the founders. These results are similar to a previous observation using a SNP indicator, in which no alleles were lost for approximately 1100 SNP loci despite two different selection schemes including a 5% selection intensity^24^. The Shannon Index (Fig. 1) showed a slightly lower, non-significant, value after roguing (Fig. 1). However, the expected heterozygosity for the five seedlots (0.29) was almost twice the heterozygosity of a white spruce seed orchard in Saskatchewan, Canada, with *H*_*e*_ = 0.16^30^, using allozyme markers, and a *Picea glauca x P. engelmannii* seed orchard in British Columbia, Canada, with an *H*_*e*_ = 0.20^31^, also using allozyme markers. Other closely related species showed higher expected heterozygosity, such as *Picea abies* (0.72)^32^ using microsatellites, and *Pinus thunbergii* (0.34–0.50)^33^ using SNPs. Overall, however, comparisons among studies can be difficult given that different types of genetic markers were used which can lead to different estimates of heterozygosity, in addition to the inherent differences in base genetic diversity levels among conifer species. In our study, expected (*H*_*e*_) and observed heterozygosity (*H*_*o*_) were similar among all years (~ 0.3), in contrast to studies conducted in *Pseudotsuga menziesii*, where *H*_*o*_ varied between 0.44 and 0.78, which was attributed to the inclusion of new parents^13^. Although low or no inbreeding has been shown in several seed orchards, such as *Picea abies* (0–0.04)^34^, and *Juglans nigra* (0.10)^35^, exceptions do exist, however, such as those observed in *Pseudotsuga menziesii* (0.22)^36^. In contrast, we found negative values of *F*_*i*_ for the 2018 seedlot and the progeny trials (− 0.042 and − 0.026, respectively) (Fig. 1). The elimination of 65% of the clonal seed orchard thus resulted in an excess of heterozygotes in the 2018 seedlot, as indicated by a negative *F*_*i*_ value. It is not clear if this value reflects a positive selection for heterozygotes in the residual population, which might be possible if the SNPs are linked to loci under selection^24^. Previous genetic diversity studies of *Khaya grandioliola* also revealed such an excess of heterozygotes, with a relatively large negative inbreeding coefficient (*F*_*i*_ =  −0.31) estimated from 53 superior trees from three provenances in Brazil^37^. Furthermore, if the inbreeding coefficient is negative after roguing, it would suggest than more homozygotes than heterozygotes have been removed, which indeed could be a result of slight inbreeding depression. A previous study in an eastern white spruce breeding population showed a decrease in height growth associated with a slight increase in relatedness (*F*_*i*_ = 0.1)^25^. Conversely, aggressive roguing increased the level of relatedness in the 2018 seedlot, with 24% and 2% more half- and full-sibs, respectively, when compared to the 2009 seedlot before roguing (Table 1). Although founders showed no half- or full-sib trees (Table 1), the identification of second cousins (31.5%) indicates that not all trees were equally spaced within the sampled stands. Seed orchards may be designed to maintain the genetic base of the parents producing deployable seedlots; however orchards also often have a different genetic arrangement when compared with natural stands^38^, which may lead to a significant increase in relatedness. In Alberta^8^, every seedlot must meet an *Ne* level ≥ 18, however, combined with uneven contributions and increased relatedness within these seedlots, policy requirements may be difficult to meet. Furthermore, when developing new orchards, parent material should be considered from several sources including the original breeding population, superior genotypes from the existing orchard, forward selections from the progeny trials, and if genomic selection is adopted, progeny from controlled crosses from either within the orchard or progeny trial trees.

### Severe roguing led to a large decrease in ***N***_***e***_ with a small increase in genetic gain

In this study, the use of genomic profiles obtained by SNP genotyping indicated a high *N*_*e*_ for founders (*N*_*e*_ = 500) of the G1 white spruce breeding program by including a correction for the number of potential fathers in addition to mothers (N = 151) (Table 1). Furthermore, all four seedlots assessed prior to roguing maintained an *N*_*e*_ between 59 and 166 (Table 1). Our numbers are higher than those seen in a comparable *Picea abies* seed orchard, which exhibited an *N*_*e*_ = 170 for founders (threefold lower than the present G1 orchard), and *N*_*e*_ values between 22 and 29 for a seed orchard with 60 parents (twofold lower than the present G1 orchard)^32^. The ratio of effective versus actual population size (*N*_*ratio*_) can also be used as an indicator of residual genetic diversity. In our study, the *N*_*ratio*_ before roguing was 0.6 (Table 1). Previous reports have shown similar numbers for orchards of *Picea abies* (*N*_*ratio*_ = 0.6)^32^, and *Pseudotsuga menziesii* (*N*_*ratio*_ between 0.72 and 1.1 for two different seedlots)^12,13^. Classical breeding theory suggests a positive linear relationship between effective population size and long-term response to selection for polygenic traits^39^. In a study comparing different breeding programs of *Triticum aestivum,* during in a 20-year period, Gorjanc et al.^40^ showed a linear relationship between *N*_*e*_ and genetic gain efficiency, with the highest gain efficiency obtained using controlled crosses exhausting all genetic diversity. In contrast, backward selection applied to the present spruce clonal seed orchard resulted in twice the gain in height (from 2.5 to 5%) but reduced *N*_*e*_ almost eightfold in the 2018 seedlot following roguing (from *N*_*e*_ = 96 to *N*_*e*_ = 12) (Table 1, Supplementary Fig. S5), suggesting that fewer trees and less pollen diversity remained in the orchard. Severe roguing resulted in only a 2–5% increase in gain, which is similar to values reported for *Pinus sylvestris* and *Pinus taeda* when removing the poorest clones from the breeding program^41,42^. The impact of genetic loss with a reduced *N*_*e*_ indicates that the breeding program needs to include forward selections, to increase the *N*_*e*_, and move forward and develop a new second generation orchard. The 50/500 rule raised four decades ago stated that a single isolated population with an *N*_*e*_ ≥ 50 is needed for short‐term conservation and an *N*_*e*_ ≥ 500 for long‐term conservation of genetic diversity^43^. This rule was revisited and debated recently, arguing that contemporary and less conservative calculations are needed^44^. Instead, the current suggestion is that a hypothetical conifer seed orchard holding an effective population size of 20 or greater would capture over 90 percent of the genetic variation found in natural populations^45^. Alternatively, the use of inbreeding values rather than effective population size can be used, as shown in animal breeding, to maintain genetic diversity when pedigree data is available^46^. Furthermore, the use of a minimal MAF (minor allele frequency) threshold could also be considered as a criterion when the level of genetic diversity that a breeding program is maintaining is a priority^24^.

### Impacts of severe roguing on parental contributions

Intense roguing of genotypes in seed orchards can lead to unequal parental contributions, substantially decreasing the effective population size, reducing random mating, increasing the number of full-sibs versus half-sibs and increasing the likelihood of inbreeding^39,47^. The tendency of the parental contributions of the studied seedlings exhibited an expected inverse exponential curve (Fig. 2), as reported in other studies^9,14,48^. Previous seed orchard studies have shown similar parental contributions for 80% of the seedlot, such as in *Pinus contorta* (53%)^11^, and *Pseudotsuga menziesii* (34–52%)^9,10,12,13^. In our study, severe roguing caused a large change in the seedlots’ composition: genotypes that were contributing substantially were eliminated from the clonal seed orchard, while other genotypes began contribution only after performing backward selections (Fig. 3a, b). Such a trend would significantly impact the estimated gains per year. Nonetheless, genotypic reproductive success is usually highly variable from year to year and influenced by several factors, such that calculations of parental-clone contributions based solely on seed/cone/pollen surveys in orchards are less precise than DNA marker-based analyzes^9,49^, thus resulting in imprecise estimates of genetic gain.

### Levels of pollen contamination in seed orchard seedlots

Levels of effective pollen contamination from external sources are usually variable between landscapes, years, countries, species and types of breeding programs^23,35,50,51^. In our study, the allelic frequencies were similar among groups (founders, seed orchard seedlots, progeny trials) (Table 1), and therefore the estimates of pollen contamination using genomic profiles are considered accurate. Furthermore, we did not find a correlation with statistical significance between the percentage of pollen contamination estimated using genomic profiles and those obtained with the pollen trap method (Supplementary Fig. S2). Use of pollen traps has previously shown high levels of pollen contamination in both white spruce (93%)^52^ and black spruce (*Picea mariana*) seed orchards (32–83%)^53,54^ in the region of this study, despite previous researchers showing that this method was unsuitable for measuring pollen contamination, and not practical when the amount of external contaminating pollen is very high^55,56^. Our results support these findings (Table 2) and correspond to values observed for other species: 18–29% for *Picea abies*^32^, 10–26% for *Pseudotsuga menziesii*^10,12,13,57,58^, 8–35% for *Pinus contorta*^11,49,59^, 5–52% for *Pinus sylvestris*^51,60,61^, 11–26% for *Pinus pinaster*^62,63^, and 19–41% for *Pinus thunbergii*^33^. Furthermore, as found in natural stands, which rely exclusively on wind for pollen dispersal to maintain genetic diversity and help avoid inbreeding in fragmented landscapes^64^, our study found a strong correlation (*r* = 0.92, *P* < 0.01) between wind direction and pollen contamination in the orchard seedlots produced in different years (Fig. 4b). Wind direction and high levels of pollen contamination (63–76%) have previously been found to coincide in a *Picea abies* seed orchard with continuous external pollen flow into the seed orchard^34^. The proximity of pollen source trees to seed orchards of *Quercus macrocarpa* (200 m away) and *Quercus robur* (400 m away) caused 57% and 70% contamination, respectively^65,66^. In contrast, in a *Juglans nigra* seed orchard isolated from wild stands, 14% pollen contamination was observed, compared with 57% contamination in a seed orchard close to an external source of pollen^35^. Not surprisingly, *Pinus sylvestris* orchards located 5 km and 30 km away from any external pollen source showed low rates of contamination of 6.5% and 4.3%, respectively^67,68^. Seed production in hoop houses (greenhouse-like structures), orchards located outside the native range, supplemental mass pollination and bloom delay (e.g. water cooling) have been suggested as solutions to reduce or eliminate pollen contamination^1,57^. However, when orchards are grouped together in a single location for ease of management, care must be taken to ensure weather conditions and all available silvicultural options are considered. In other species, such as commercial apples, new mass pollination techniques are being used to supplement bee pollination using a Dropcopter (see dropcopter.com) and these methods are being considered for conifers.

### Levels of pollen contamination in families from progeny trials

In addition to the contamination levels found between seedlots, we also identified a range of 7–68% in pollen contamination on a family basis within a given seedlot (Fig. 5), which is much higher compared to a range of 0–12% of pollen contamination for families of seedlots from three different years in a *Pinus sylvestris* orchard^51^. Also, the large range in pollen contamination at the individual-genotypic level, as observed in our study, could impact breeding values for each genotype, and genotype ranking based on height growth. When using open-pollinated families, although a common practice, it is risky to assume that the progenies are all true half-sibs^69,70^, as we demonstrated in this study. Reconstruction of full pedigrees from progeny trials allowed to obtain genomic BLUPs (Best Linear Unbiased Predictions) to correct the breeding values obtained from the pedigree-based models in previous studies^17,69^, which are typically biased^70^. It is clear that high rates of pollen contamination occurred, for some families in progeny trials, and this will affect the overall genetic gain calculated for the white spruce breeding program, reducing the profitability of the tree improvement program in the long-term. In a *Pseudotsuga menziesii* breeding program in British Columbia, based on open-pollinated material, an income reduction of $748,500 CAD per year, at the highest site index and a planting density of 1111 trees/ha, was projected due to pollen contamination^57^.

The current study also showed that most of the trees studied in the progeny trials were produced from 11 fathers (Fig. 5). Another recent study showed that 93% of the progeny had the same father within the seed orchard^71^. This study also showed that fathers contributing pollen in the earliest phenology stage can potentially sire more offspring in the progeny pool than fathers contributing in the intermediate or late male phenology stage^71^. Paternity testing from progeny also helped to identify male Eucalyptus that were not contributing to the offspring from a seed orchard and needed removal^72^.

The orchard manager will need to avoid using measurements from trees in the progeny trials that are not part of the population of interest, when knowing that the progeny comes from orchard collections and not from wild stands (as the case of our study), in order to get the most accurate breeding values for a particular genotype.

### Implications for orchard managers

Results from our study and others^32^ suggest that orchard managers should avoid severe roguings to prevent an increase in coancestry and risk of genetic diversity losses (Fig. 1; Table 1), without careful planning at the time of orchard establishment. Results also provide evidence of how significant the change in parental contributions can be from year-to-year when combined with the elimination of contributing genotypes (Fig. 3; Table 2). To address this, orchard managers can balance out the variability in parental contributions by mixing stored seedlots from different years using information about genotypes to ensure that the seedlots used for deployment have the desired level of genetic diversity and expected genetic gain^73^.

In addition, the use of nuclear SNP markers for pedigree reconstruction and the parental pairing revealed in our study allowed us to identify which two parents produced a given offspring in bulk seed samples without using megagametophytes, as suggested in a previous study^11^. This procedure of parental contribution is valuable (in time and cost) as it allows for the calculation of both the *N*_*e*_ and pollen contamination levels, which provides an accurate estimate of genetic worth of seed crops or effective population numbers. If use of the cones/seeds/pollen counting method will be pursued in the future, the correlation between this method and that based on genotyping and pedigree reconstruction could potentially be used to corroborate numbers for white spruce (Fig. 4a), although several studies have emphasized the need for molecular markers to obtain accurate estimates of *N*_*e*_^74–78^. Currently, SNP genotyping is very affordable for most plant breeding programs, due to a substantial reduction in cost over the last decade^79^, and because of the increase in accuracy of parental breeding values with access to the full pedigree (coancestry matrix), as seen in *Picea glauca*^17^ and *Picea rubens*^80^.

An *N*_*e*_ of 18 is a critical requirement to meet policy regulations in Alberta, Canada. As companies work to increase their gain as much as possible (through roguing) while still meeting *N*_*e*_ requirements, the difference between 12 and 18 is the difference between approval to deploy and reforest or not. Although in British Columbia, Canada, the *N*_*e*_ requirement is 10, that is not the case in Alberta. If the *N*_*e*_ is estimated to be 12 for a particular seedlot, in Alberta, then a company would have to blend seedlots from earlier years, or wait until another crop could be harvested to meet the policy requirements.

Moreover, a distance of 600 m was proposed by Khanduri^81^ as a minimum requirement to prevent contamination by pollen in the management of seed orchards. Nevertheless, contrasting contamination numbers from different orchards and species have shown that the use of physical distance alone is controversial, given that recommended distances have generally been insufficient to ensure low contamination levels^34,65,66^. Although the clonal seed orchard we studied is located far from natural stands of white spruce, in 1998, another white spruce orchard for an adjacent region was established 1 km west (Supplementary Fig. S5), despite an expectation of a prevailing southwest wind direction during the pollination period^15^. As expected, the average wind direction between 15 and 31 May, when the pollen is at maximum dispersal in Alberta, ultimately showed a correlation of 92% with pollen contamination levels in the Region G1 seedlots, during the five years of this study (Fig. 4b). This correlation shows that a minimum of 11 days with the wind direction, coming from a relatively close (1000 m away) pollen source to the target orchard during peak pollen production and conelet receptivity, can result in up to 50% pollen contamination (Table 2). These results suggest that a 1 km distance is not sufficient to reduce pollen contamination to acceptable/desirable levels based on wind direction and a lack of interception by other silvicultural or physical means (e.g. type or size of windbreaks). It is vital that tree improvement programs consider orchard location with considerable care, using information about wind speed and direction, distance between orchards, altitude and barriers, to analyze the economic trade-offs of implementing alternative silvicultural practices such as tents over the trees^60^, supplemental mass pollination^82^, bloom delay^1^, or using a mixture of these methods to reduce pollen contamination. A further unintended consequence of pollen contamination, from an orchard producing seed destined for a different region, could also be the production of maladapted seedlings.

The commonly held view by orchard managers, of reduced pollen contamination with increasing age and crown volume of seed orchards, does not always occur. Levels of pollen contamination can be quite high, even in mature seed orchards, as found in a *Pseudotsuga menziesii* seed orchard over a ten-year assessment period^83^. Consequently, if a reduction in pollen contamination can be ensured for each crop produced, the genetic worth of the seedlots will be increased. Combined with the roguing, the 3-year cycle of crown topping, also applied in the studied orchard, allows for greater area around each tree, more light, potential access to moisture and higher air flow. These factors can all affect conelet development and exposure to contaminating pollen penetrating deeper into the orchard.

Lastly, since in practice, the principle of random mating is not applicable in operational plant breeding, simulations are needed to carefully design breeding programs^79^. For example, simulations using the POPSIM software^84^ in a *Pinus pinaster* improvement program, which was designed to maximize both gain and genetic diversity, generated a strategy of performing 150 controlled-crosses to obtain 100 progeny per cross as the most effective approach, but also the most time-consuming approach^18^. As an alternative, the option of using a polycross mating design together with paternity recovery using genotyping, is emerging as the preferred strategy to increase gain, and retain the desired effective population size with only a small decrease in male contributions, thereby allowing tree improvement programs to advance more quickly^17,18,80,85,86^. Although our data does not address the advantages of using a polycross directly, understanding the options and impact of different breeding strategies is critical to the rapid advancement of any long-lived tree species program.

## Methods

### Study area

The Region G1 white spruce Controlled Parentage Program (CPP) began in 1979 with parent selections from wild stands in Alberta, Canada. The first-generation seed orchard (G351) and the first progeny trials (G135) were established in 1988 (Phase 1). The second series of progeny trials (G365) were established in 2005 (Phase 2). The seed orchard under study (G351) is a clonal seed orchard, thus established from ramets or grafts collected from the original parent trees from wild stands, rather than a seedling seed orchard which is established using seeds from the wild stand parent trees. Initially, the clonal seed orchard had 151 ‘founders’ selected from wild stands (with ~ 12 ramets per selected parent tree (clone)), and based on the progeny trials growth measurements, the Phase I clonal seed orchard have received two small and one severe roguing so far. The first roguing removed six genotypes (all ramets) and occurred in fall of 2009 and the second roguing removed eight genotypes (all ramets) and occurred in spring of 2010. The third and last roguing removed 84 genotypes and occurred in spring of 2018, leaving 53 genotypes and representing a total elimination of 65% of the orchard by summer 2018^15^. The clonal seed orchard is located near Grande Prairie, Alberta (lat. 55°03′51″ N, long. 119°16′24″ W, 720 elevation) and the four Phase 2 progeny trials (G365 A-D) are located in northwestern Alberta. The genetic worth (GW) of the 2007 clonal seed orchard crop was 2.5% (based on height at rotation), and increased to 5.0% (height at rotation) after the three roguings^87^. Trees in the G1 orchard are managed on a 3-year cycle of crown topping to keep them within a manageable size for cone harvesting. Each topping has an unknown impact on cone production.

### Genetic material, collection and DNA extraction

A random sample of 105 open-pollinated bulk seeds from five different years were used for this study. The Alberta Tree Improvement and Seed Centre (ATISC) governmental facility supplied stored seedlots corresponding to years 2003, 2005, 2007 and 2009, and all of them were collected in summer time and before any roguing. Seedlings tested from the 2018 seedlot was randomly selected from the operational seedlot collected in the fall of 2018, and reflects the cumulative effect of the three roguings (two small and one severe). A total of 525 seeds were sown in January 2019 at a commercial forest nursery (Bonnyville, Alberta, Canada), and seedlings were harvested for DNA extraction five months later. Moreover, progeny from the top 10 open-pollinated families (collected from the clonal seed orchard in 2003 and planted in 2005) of the Region G1 white spruce CPP were selected for genotyping to assess genetic diversity and pollen contamination within families. The 10 families selected were from a total of 341 families (originally established in the G3645 progeny trial), 71 of which originated from individual genotype collections in the clonal seed orchard. Ranking of families from the progeny trial is based on the breeding values for height (%) at rotation. Newly flushed needles were collected from 152 trees at the G365 (site B) progeny trial and 176 trees at the G365 (site D) progeny trial, with approximately 33 progeny trees sampled per family. The 151 founders were sampled at the white spruce clone bank (G218) at ATISC (Smoky Lake, Alberta, Canada). Current year needles from parents and progeny trees were collected between May and June 2019 using pole pruners and scissors. Tissue was placed into plastic bags, stored at 4 °C before transportation to the extraction facility within two days of collection. DNA was extracted at InnoTech Alberta using the Qiagen DNeasy Plant Kit (Mississauga, Ontario, Canada) and quantified using a Nano-Drop N-1000 spectrophotometer (Thermo Fisher Scientific, Waltham, MA, USA).

### Genotyping assay

A total of 1004 DNA samples and one control sample per 24-well plate (total of 44) were genotyped using an Infinium iSelect SNP array (Illumina, San Diego, CA) previously designed from eastern white spruce material and gene SNPs^88,89^. The chip used in this study was composed of 5308 biallelic SNPs representing as many distinct loci spread along the 12 linkage groups of white spruce^17^. Genotyping was conducted by Neogene Canada (Edmonton, Alberta, Canada). In order to have an optimal parental assignment and pollen contamination assessment, we discarded a total of 555 SNPs, of which 547 were either monomorphic, paralogs, presented low or null signal, or were multilocus from visual inspection using GenomeStudio 2.0, while eight others had a MAF < 0.01, absolute value of fixation index |*F*_*e*_| ≥ 0.50 or had an average call rate < 85% (Supplementary Table S1). Valid SNPs (4753) had an average call rate per SNP and per sample of 99.5%, average MAF of 0.22, and average *F*_*e*_ of − 0.01. The reproducibility rate of the assay showed a value of 99.99%, which was calculated with the two control samples (Supplementary Table S1). Two subsets consisting of 2000 SNPs each (SNP subset 1 and 2) were assembled from random draw without replacement. The two subsets did not show differences when performing the diversity and parentage analysis, and showed similar inbreeding coefficients, because inbreeding affects the entire genome at the same time, for all neutral population processes including mating systems, natural population structure and IBD (identity by descent), migration and demography including bottleneck effects. Thus, sampling of even a small number of markers (hundreds) would also result in a not significantly different inbreeding coefficient. Consequently, we performed all of the subsequent analysis using subset 1. Pedigree reconstruction can be accurately done for thousands of individuals with a minimum of 60–100 SNPs with moderate to high MAF values^90^, so using 2000 SNPs was considered to be sufficient for this study.

### Diversity analysis

The Shannon Index (*I*), observed (*H*_*o*_) and expected heterozygosity (*H*_*e*_), inbreeding coefficient (*F*_*i*_), and coancestry coefficient (*Ɵ*) were calculated using the GenAlEx program, v6.5^14,91^ and the SNP subset 1 (2000 SNPs, corresponding to 2000 distinct gene loci). The inbreeding coefficient was calculated as *F*_*i*_ = (Mean *He*—Mean *Ho*) / Mean *He*. Effective population size (*N*_*e*_) is defined as the census size of a population of unrelated, non-inbred individuals with equivalent gene diversity^92^, and measures the rate of genetic drift and inbreeding^93^. Effective population size was calculated using two methods. The first method was based on number of cones produced by genotype in a clonal seed orchard, with the formula *N*_*e*_ = 1/(Σ*p*_*i*_^2^), (i = 1, 2, …, n), where *p*_*i*_ = the proportional contribution (number of cones) of genotype *i*, n = number of genotypes^8,92,94^ (standard assessment of effective population size in many orchards is through cone counts, collected by genotype, and is conducted in the absence of genotypic data which is typically not available for cross validation). The second method was based on genomic profiles with SNPs, calculated with the formula *N*_*e*_ = 0.5/*Ɵ*^17,95,96^. The relatedness matrix (pairwise relationships between offspring) obtained with the GenAlEx program allowed us to group the relatedness levels of progeny following the identity coefficients traditionally used in genetics, such as unrelated (*Ɵ* = 0), second cousins (*Ɵ* = 1/16), first cousins (*Ɵ* = 1/8), half-sibs (*Ɵ* = 1/4), full-sibs (*Ɵ* = 1/2) and clones (ramets with the same genotype) (*Ɵ* = 1)^97^. To estimate the variability of gene diversity among and within groups (founders, seed orchard seedlots, progeny trials), we performed an AMOVA (Analysis of Molecular Variance) based on the *Φ*_*PT*_ statistic (measure of the degree of genetic differentiation)^98^, using 2000 SNPs, 1004 samples, eight groups (founders, five seedlots, two progeny trials) and 1000 permutations. Site B and site D progeny trials did not show significant differences regarding genetic diversity, so the 328 trees were treated as one group, called “Progeny trials” throughout the text, for simplification purposes.

### Parentage assignment, pedigree reconstruction and pollen contamination

The parentage analysis was conducted on each offspring from the different groups (bulked seedlots and progeny trees) using CERVUS 3.0.7, with an assignment probability of 95%, a conservative genotyping error rate of 0.0001 (Supplementary Table S1), and SNP subset 1 (2000 SNPs). This likelihood-based program outputs the two most likely parents, allowing for the reconstruction of half- and full-sibling families^99,100^. In seed orchard crops, it is often sufficient to know the two parents without knowing their gender (DNA extractions from maternal megagametophytes were not performed), because diversity parameters such as effective population size, pollen contamination and genetic gain can still be estimated^11^. CERVUS was run using the “parent pair-sexes unknow” analysis. For the five seedlots, parents were called “parent sex1” and “parent sex2”, and for the progeny trials, parents were called either “mother” or “father”. For each offspring, parent sex1 (mothers of trees from the progeny trials) with a positive LOD score was accepted, and parent sex2 (fathers of trees from the progeny trials) was confirmed when the delta score was significant. The delta score is the difference in LOD scores between the first and second most likely parent pair, and it was calculated with a simulation of 10,000 offspring and assuming that 50% of candidate parents were sampled^14,17^. The results obtained with CERVUS were confirmed with COLONY 2.0.6.5., using the default options. The parental contributions before any roguing (seedlots years 2003, 2005, 2007, 2009, total offspring = 420) were combined and compared with the contributions after the three roguings (seedlot year 2018, total number of offspring = 105) with percentages based on total offspring. Mating dynamics of the five seedlots together and the 10 progeny families were performed based on the number of offspring that each mother (parent sex1) and father (parent sex2) produced, using Excel. Pedigree reconstruction for the progeny trial trees was plotted using the ggnetwork package (version 0.5.8) in the R environment.

### Pollen contamination assessment

In general, external pollen contamination in seed orchards is measured to adjust genetic gains. Pollen contamination (*PC*) of the white spruce orchard was estimated using two methods. The first method consisted of using pollen monitors (traps) outside and inside the orchard, and a surrogate species (e.g. pine pollen), with the formula *PC* = (*WS*/*XS*)*(*XT*/*WT*), where *WS* = within-orchard pollen level of surrogate species, *XS* = outside orchard pollen level of surrogate species (if *WS*/*XS* ratio is > 1, use 1), *XT* = outside orchard pollen level of white spruce, *WT* = within-orchard pollen level of white spruce^8^. The second method for the pollen contamination assessment consisted of using genomic profiles for parental assignment: an offspring was labeled as ‘pollen contamination’ when the delta score was not significant, which means a mismatch occurred between candidate parents^11^.

### Correlations for effective population size and level of pollen contamination

A correlation value was estimated between *N*_*e*_ means based on cones and *N*_*e*_ based on genomic profiles for each seedlot year studied. In addition, correlation values were estimated between levels of pollen contamination based on genomic profiles and climate parameters (means of temperature, relative humidity, wind speed, wind direction) between 15 and 31 May of each studied year. Weather data used in this study corresponded to the ‘Grande Prairie A’ weather station, which is the closest station to the orchard located 30 km away. For years 2003–2018, climate data was obtained from www.climate.weather.gc.ca, and for 2019–2020, data was obtained from www.acis.alberta.ca. Pearson’s correlation (*r*) coefficients were estimated in the R environment and assumed to be significant at *P* < 0.05. Equations, predicted values and plots were fitted using Excel.

## Supplementary Information

Supplementary Information.
