# Supplementary material for: SNP-based analysis reveals unexpected features of genetic diversity, parental contributions and pollen contamination in a white spruce breeding program
Source: Sci Rep. 2021 Mar 2;11:4990. doi: 10.1038/s41598-021-84566-2 (PMC7925517; doi:10.1038/s41598-021-84566-2)

**Title:** SNP-based analysis reveals unexpected features of genetic diversity, parental contributions and pollen contamination in a white spruce breeding program

**Authors:** Esteban Galeano^*1^, Jean Bousquet^2^, Barb R. Thomas^1^

**Affiliation**: ^1^Department of Renewable Resources, 442 Earth Sciences Building, University of Alberta, Edmonton, Alberta, Canada. T6G 2E3. ^2^Department of Wood and Forest Sciences, Pavillon Charles-Eugène Marchand, Université Laval, Quebec, Canada. G1V 0A6.

***Corresponding Author**

**Emails:** Esteban Galeano ([galeanog@ualberta.ca](mailto:galeanog@ualberta.ca)), Jean Bousquet ([jean.bousquet@sbf.ulaval.ca](mailto:jean.bousquet@sbf.ulaval.ca)), Barb R. Thomas ([bthomas@ualberta.ca](mailto:bthomas@ualberta.ca)).

**SUPPLEMENTARY INFORMATION**

Supplementary Table S1. Statistics of different parameters for a white spruce SNP chip containing 5308 SNPs, after genotyping 1056 samples and 44 controls, with and without failed SNPs. SNPs were considered as “failed” (and discarded) by visual inspections (low or null signal, monomorphic, paralogs and multilocus SNPs) using GenomeStudio. SNPs were “filtered” (and discarded) if they had a minimum allele frequency (*MAF*) < 0.01, absolute value of fixation index |*Fe*| ≥ 0.50 or average call rate <85%. Call rate is shown per SNP and per sample.

| **Parameter** | **Including  failed SNPs** | **Without failed and monomorphic**  **SNPs** | **Without filtered SNPs** | **For pedigree reconstruction** |
| --- | --- | --- | --- | --- |
| Number of SNPs | 5,308 | 4,761 | 4,753 | ~2,000 |
| Number of samples | 1056 (with  controls) | 1049 (with  controls) | 1049 (with  controls) | 1001 (without  controls) |
| **Means of *MAF*** |  |  |  |  |
| All SNPs | 0.194 | 0.217 | 0.217 | 0.217 |
| *MAF* ≥ 0,01 | 0.203 | 0.217 | 0.217 | 0.217 |
| *MAF* ≥ 0,05 | 0.204 | 0.228 | 0.228 | 0.228 |
| **Means *F_e_*** |  |  |  |  |
| All SNPs | -0.0092 | -0.0104 | -0.0095 | -0.0095 |
| SNPs with -0,5<*F_e_*<0,5 | -0.0084 | -0.0095 | -0.0095 | -0.0095 |
| **Means call rate per SNP** |  |  |  |  |
| All call rates | 88.94 | 99.48 | 99.48 | 99.48 |
| Call rates ≥ 85% | 99.14 | 99.48 | 99.48 | 99.48 |
| Call rates ≥ 90% | 99.14 | 99.48 | 99.48 | 99.48 |
| **Means call rate per sample** |  |  |  |  |
| All call rates | 88.94 | 99.48 | 99.48 | 99.81 |
| Call rates ≥ 85% | 89.55 | 99.80 | 99.80 | 99.81 |
| Call rates ≥ 90% | N/A | 99.81 | 99.81 | 99.81 |
| **Reproducibility rate** | **Number of calls** | **Number of mismatches** | **Reproducibility rate** |  |
| Control 1 (20 samples with 4761 SNPs) | 95,240 | 10 | 99.99% | - |
| Control 2 (19 samples with 4761 SNPs) | 90,459 | 5 | 99.99% | - |

Supplementary Table S2. Summary AMOVA (Analysis of Molecular Variance) table. *P* values are based on *Φ_PT_* statistics, using genomic profiles with a set 2,000 SNPs, 1,004 samples, 8 groups (founders, five seed orchard seedlots, two progeny trials) and 1000 permutations. Df=degree of freedom, SS=sum of squares, MS=mean squares, EV=estimate of variance, ***Φ_PT_*** =estimate of population genetic differentiation, %=percentage of total variation.

| **Source** | **Df** | **SS** | **MS** | **EV** | **%** | ***Φ_PT_*** | ***P* value** |
| --- | --- | --- | --- | --- | --- | --- | --- |
| **Among groups** | 7 | 9326.14 | 1332.31 | 8.41 | 3% | 0.028 | <0.001 |
| **Within groups** | 996 | 287129.0 | 290.03 | 290.03 | 97% |  |  |
| **Total** | 1,003 | 296455.21 |  | 298.44 | 100% |  |  |

Supplementary Table S3. Estimates of relatedness levels (%) among individuals within half-sib families from the progeny trials, after disaggregating the coancestry coefficients obtained after pedigree reconstruction using genomic profiles.

| **Relatedness  level** | **Half-sib family from progeny trials** | | | | | | | | | |
| --- | --- | --- | --- | --- | --- | --- | --- | --- | --- | --- |
|  | **F91** | **F132** | **F138** | **F755** | **F966** | **F1001** | **F1002** | **F1004** | **F1045** | **F1047** |
| Unrelated | 0% | 5% | 10% | 0% | 10% | 6% | 0% | 0% | 11% | 8% |
| 2nd cousins | 0% | 1% | 1% | 0% | 2% | 1% | 0% | 0% | 0% | 4% |
| 1st cousins | 1% | 5% | 12% | 6% | 8% | 5% | 10% | 5% | 2% | 6% |
| Half-sibs | 89% | 77% | 73% | 83% | 80% | 85% | 85% | 89% | 86% | 77% |
| Full-sibs | 10% | 12% | 4% | 11% | 1% | 4% | 5% | 6% | 1% | 5% |

Supplementary Figure S1. Three-dimensional representation of the mating dynamics in the G1 white spruce orchard based on 5 years of assessment. Diagram was based on 420 seedlings and 151 parents for years 2003, 2005, 2007, 2009 (before roguing), and 105 seedlings and 53 parents for year 2018 (after roguing).

**
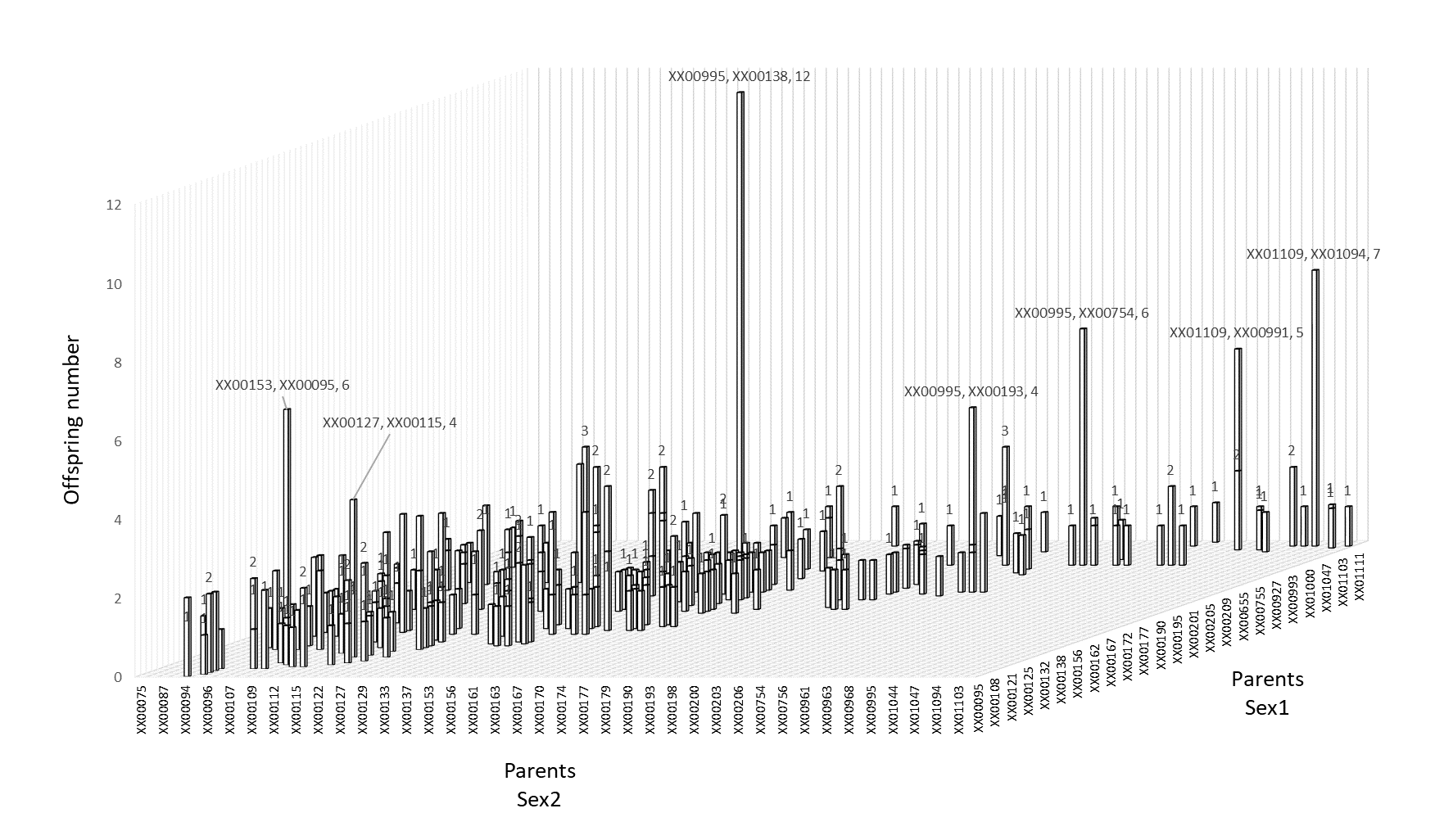
**

Supplementary Figure S2. Correlation plot (Pearson’s) showing trendline (green line), *r* value and *P* value (gray square) for the levels of pollen contamination (%) using genomic profiles versus pollen traps.

Supplementary Figure S3. Pearson’s correlations between levels of pollen contamination from genomic profiles (%) and climate parameters (means between 15-31 May of five different years). Weather data for 2003-2018 was obtained from [www.climate.weather.gc.ca](http://www.climate.weather.gc.ca), and for 2019-2020 from [www.acis.alberta.ca](http://www.acis.alberta.ca) from the ‘Grande Praire A’ weather station.


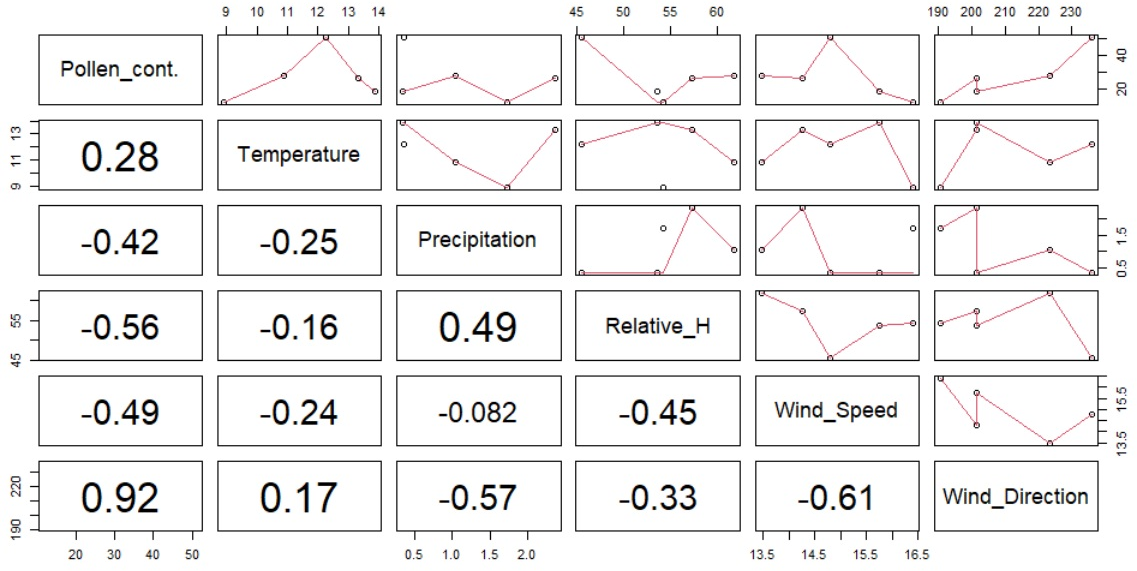


Supplementary Figure S4. Three-dimensional representation of the mating dynamics in two 15-year-old progeny trials, part of the Region G1 white spruce improvement program. Diagram was based on 10 families (328 trees) and 151 parents.

**
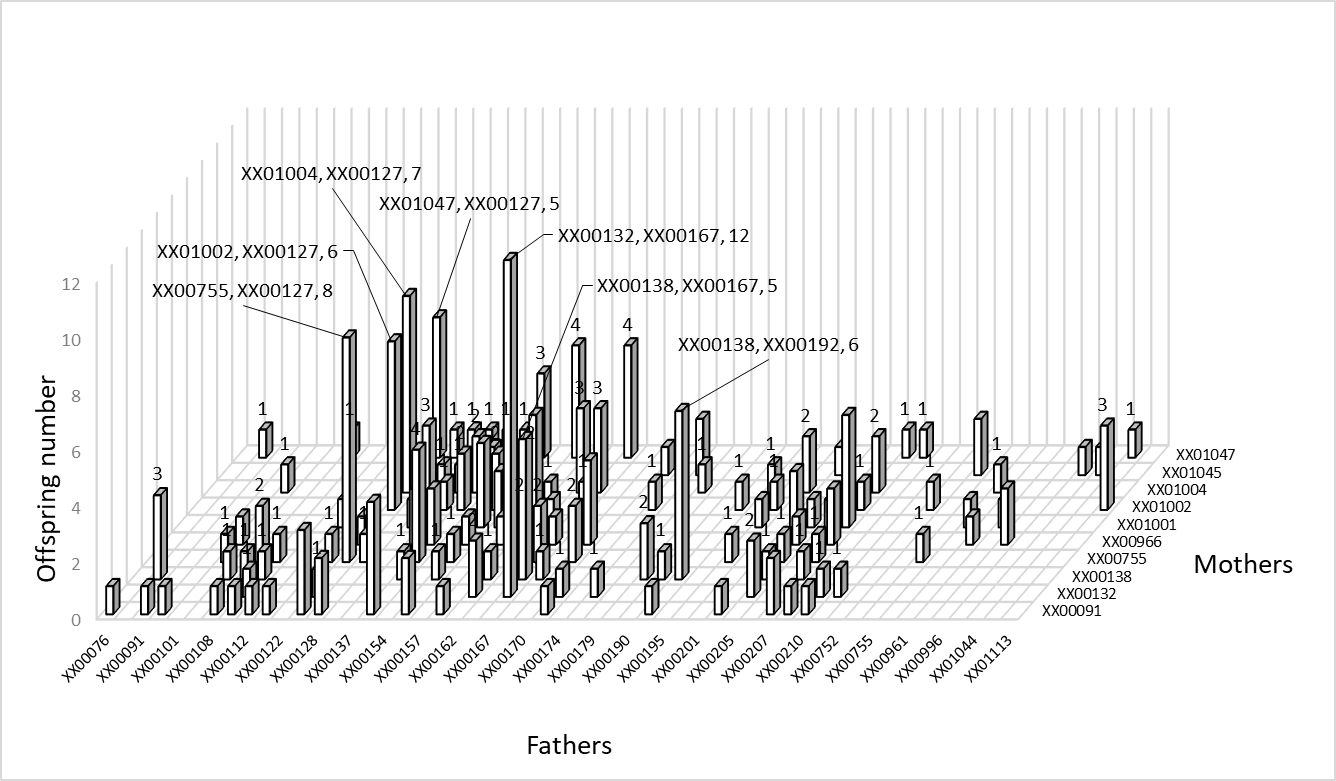
**

Supplementary Figure S5. Radar chart showing genetic gains (% height), coancestry coefficient (*Ɵ*) and effective population size (*N_e_*) calculated using numbers of cones and genomic profiles with a set of 2,000 SNPs, before and after roguing the G1 white spruce orchard.


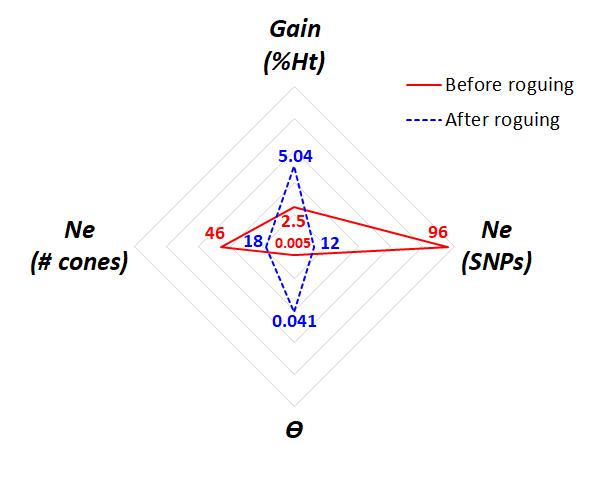


Supplementary Figure S6. Top view diagram of the orchards at HASOC (Huallen Seed Orchard Company), Grande Prairie, Alberta, Canada (lat. 55°03’51” N, long. 119°16’24” W, 720 elevation). The target orchard of study and the putative contamination source orchard are highlighted with blue and red squares, respectively. The other orchards present in the site are highlighted with black squares.


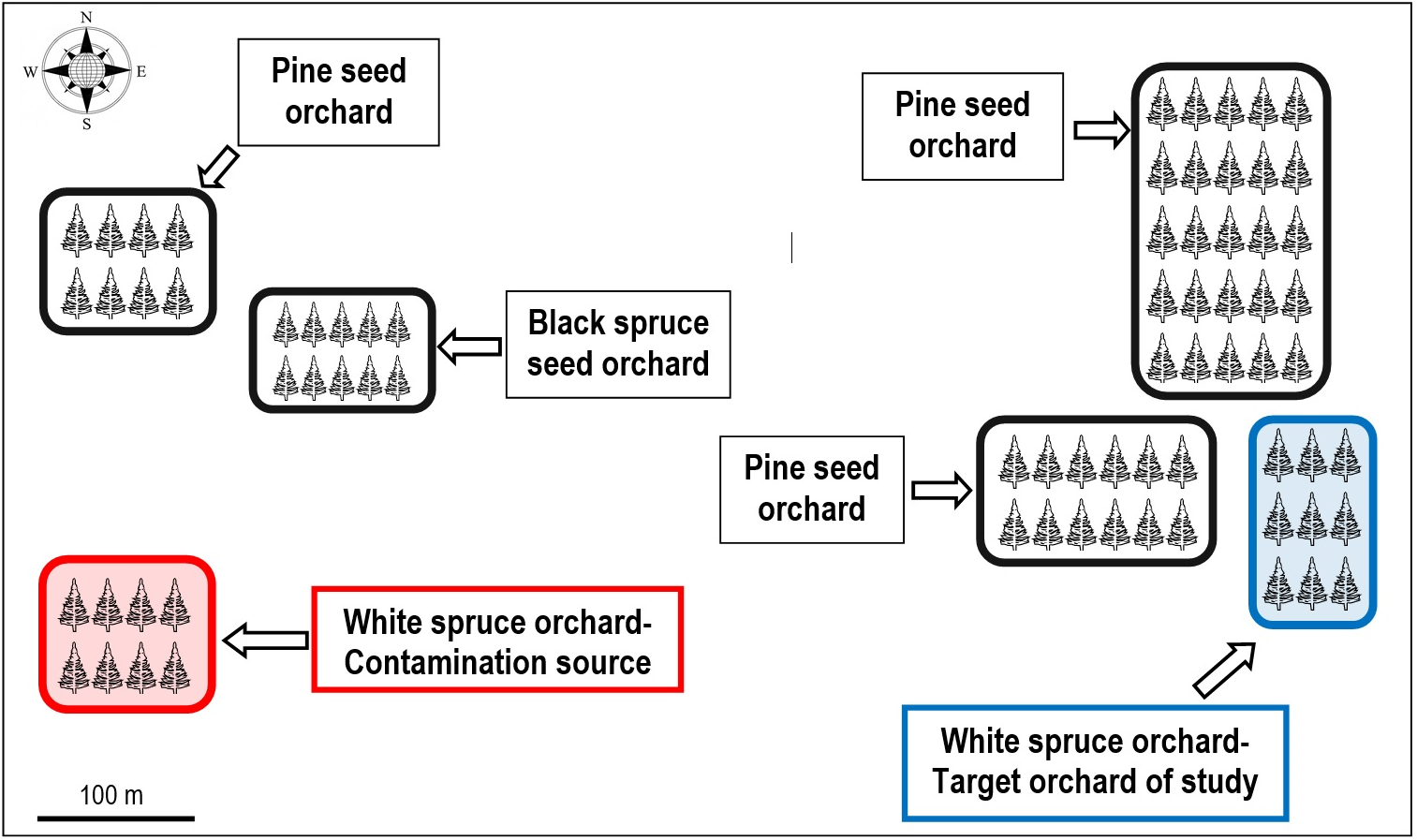

Supplement: Supplementary file 1 — Supplementary Information. [file 41598_2021_84566_MOESM1_ESM.docx]
